# Colonoscopy report generation using voice recognition system

**DOI:** 10.1055/a-2845-3517

**Published:** 2026-04-20

**Authors:** Ryosuke Kawagoe, Yutaka Saito, Yasuhiko Mizuguchi, Mai Ego Makiguchi

**Affiliations:** 1 68380 Endoscopy Division, National Cancer Center Hospital, Tokyo, Japan

Accurate and efficient documentation is essential in colonoscopy reporting; however, the process is often time-consuming and prone to errors due to manual data entry. In recent years, voice recognition has been developed to address these issues 1 2 3 .

We evaluated the utility of a voice recognition system, EVAS (KENKONE, Taiwan), for colonoscopy reporting ( Video 1 ). A pilot study was conducted to compare the report generation time between the EVAS and conventional typing. Twenty-one cases were analyzed, including patients undergoing screening, surveillance, diagnostic evaluation, or endoscopic treatment. Cases with familial adenomatous polyposis, suspected GVHD, enteritis, or patients under 20 years were excluded.

Colonoscopy report generation using a voice recognition system (EVAS).Video 1

Five experienced endoscopists measured the time required to enter endoscopic findings per report. The mean report generation time using EVAS was 29 seconds, a 46.3% reduction compared to 56 seconds with manual typing.

**Fig. 1:**
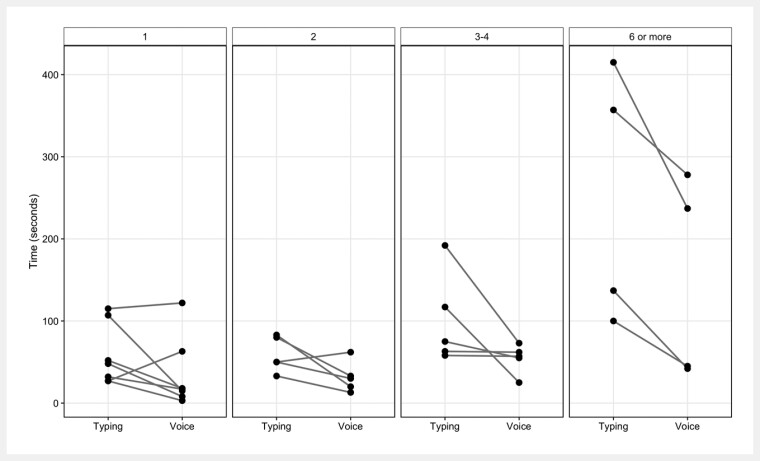
Results of the pilot study. Patients were divided into four groups based on the number of findings: 1 finding, 2 findings, 3–4 findings, and 6 or more findings. The report generation time tended to decrease with a greater number of findings in the EVAS group compared to the typing group.

The colon often contains multiple polyps, and the report generation time tends to decrease with a greater number of findings and interventions in the EVAS group compared to the conventional typing group ( Fig. 1 ).

Our findings suggest that voice recognition can reduce the report generation time, particularly when numerous findings are recorded. However, issues such as recognition accuracy and integration with filing systems remain. Further prospective studies are planned to evaluate reporting accuracy and clinical applicability.

Endoscopy_UCTN_Code_TTT_1AU_2AD
